# Gut mycobiome in cardiometabolic disease progression: current evidence and future directions

**DOI:** 10.3389/fmicb.2025.1659654

**Published:** 2025-10-09

**Authors:** Xiaoyu Wei, Zixin Guo, Jingyang Wang, Dan Gao, Qiang Xu, Shengyu Hua

**Affiliations:** ^1^College of Traditional Chinese Medicine, Tianjin University of Traditional Chinese Medicine, Tianjin, China; ^2^Department of Cardiology, Second Affiliated Hospital of Tianjin University of Traditional Chinese Medicine, Tianjin, China

**Keywords:** gut mycobiome, fungi, gut microbiota dysbiosis, cardiometabolic diseases, fungal metabolites

## Abstract

Cardiometabolic diseases (CMD) are a cluster of complex syndromes characterized by cardiovascular damage resulting from metabolic dysregulation; however, their underlying mechanisms remain unclear. Recently, CMD research has paid considerable attention to the gut microbiota, though the emphasis has been on bacterial communities, and the gut mycobiome’s role is still not well understood. Hence, this review consolidates information on the correlation between the gut mycobiome and CMD and examines how the gut mycobiome may play a role in CMD progression. Accumulating evidence indicates that gut mycobiome dysbiosis, particularly the aberrant expansion of specific fungal genera such as Candida and Saccharomyces, is closely associated with the development and progression of cardiometabolic diseases. This association is primarily mediated through multiple mechanisms. For instance, fungal metabolites (enzymatic derivatives, alcohol) enhance intestinal lipid absorption, accelerate hepatic steatosis, and trigger systemic insulin resistance. Meanwhile, dysregulated cross-kingdom interactions disrupt intestinal barrier function, leading to endotoxemia and vascular inflammation, thereby promoting atherosclerosis. Additionally, fungal pathogen-associated molecular patterns activate the immune-metabolic axis, resulting in adipose tissue inflammation and glucose dysregulation. These pathways interact synergistically, collectively exacerbating metabolic dysfunction and cardiovascular damage. We also outline strategies targeting the gut mycobiome as a potential therapeutic approach for cardiometabolic diseases. By integrating current state-of-the-art insights, this review provides a critical reference for the development of novel mycobiome-based interventions in cardiometabolic disease management.

## 1 Introduction

Vertical transmission from the mother during the perinatal period initiates colonization of the human body by microorganisms (Wang et al., 2020). These microorganisms continue to adapt and evolve as the host matures, leading to the formation of a more stable adult microbiota (Kumbhare et al., 2019). Every person has a diverse and interlinked collection of microbial communities (Costello et al., 2009). Recent studies have confirmed that the partnership between microbiota and humans is vital for developing and healing diseases (Aggarwal et al., 2023). These microscopic entities are as numerous as the human cells they inhabit (Sender et al., 2016), primarily residing in the distal section of the gastrointestinal tract.

The gut microbiota comprises various microorganisms, including bacteria, archaea, fungi, and viruses, with bacteria being the predominant group (Sender et al., 2016). These gut microorganisms contribute to the host’s energy metabolism through their metabolites and byproducts and are essential for developing the host’s immune system (Carmody and Bisanz, 2023; Arnaiz-Villena et al., 2024), which supports physiological functions. Beyond these roles, the gut microbiota also exerts endocrine regulatory functions, modulating hormones such as leptin, ghrelin, and cortisol, thereby influencing host energy balance. It plays a crucial role in maintaining intestinal barrier integrity and produces diverse metabolites—including short-chain fatty acids (SCFAs), branched-chain fatty acids, and phenolic compounds—that can enter systemic circulation and affect cardiovascular physiology via anti-inflammatory effects, barrier enhancement, and signaling regulation (Datta et al., 2024; Zhang et al., 2025). Researchers have observed that disturbances in gut microbial communities are associated with various diseases, including neurodegenerative, cardiovascular, metabolic, and gastrointestinal disorders (Chen et al., 2021; Wei et al., 2025). Traditionally, investigations into how gut microbial populations relate to human health or illness have primarily centered on bacteria. However, the emergence of metagenomic high-throughput sequencing technologies has led to a greater appreciation of the gut mycobiome’s role in affecting the human host.

Although the gut mycobiome comprises only about 0.1% of the overall intestinal microbiota in healthy adults, it is a critically important component (Nash et al., 2017). The most dominant fungal taxa include *Candida*, *Saccharomyces*, *Penicillium*, and *Malassezia* (Bhunjun et al., 2024). As key intestinal components, they dynamically interact with bacterial communities and the host gut mucosal immune system, playing essential roles in regulating microbial ecosystem homeostasis (Kombrink et al., 2019; García et al., 2017). However, factors such as diet, antimicrobial use, geographical location, and age can contribute to gut fungal dysbiosis (Wu et al., 2021). The overproliferation of these opportunistic fungi directly disrupts intestinal mucosal homeostasis, leading to persistent and excessive immune activation (Huang et al., 2024), which promotes the progression of intestinal diseases. For instance, *Beauveria bassiana*-mediated upregulation of macrophage glycolytic pathways and IL-7 expression induces IL-22 secretion by type 3 innate lymphoid cells (ILC3s) through the AhR and STAT3 pathways, thereby exacerbating colorectal cancer (CRC) progression (Zhu et al., 2021). Moreover, the excessive proliferation of these opportunistic fungi not only affects the gut but can also trigger dysregulation in distal organs. For example, chronic alcohol consumption increases gut fungal abundance and promotes the translocation of fungal *β*-glucan into the portal circulation, which subsequently activates Kupffer cells via the C-type lectin-like receptor (CLEC7A) and upregulates IL-1β expression, ultimately leading to hepatocyte injury and hepatic inflammatory response (Thomas, 2017). The gut mycobiome’s association with multiple diseases has been the central theme of recent studies, particularly inflammatory bowel disease (Hsu et al., 2023), hepatic disorders (Zeng and Schnabl, 2024), autoimmune diseases (Jensen et al., 2024), neuropsychiatric conditions (Hadrich et al., 2025), and malignancies (Guglietta et al., 2025). The investigation into how the gut mycobiome might be related to cardiometabolic disease (CMD) is gaining more attention; however, this field currently remains deficient in systematic reviews and comprehensive syntheses. CMD is a clinical syndrome in which metabolic dysregulation triggers cardiovascular damage, characterized by a causal relationship between metabolic abnormalities and structural or functional cardiovascular impairments (Barteková et al., 2021; Sattar et al., 2020). This syndrome encompasses complex interactions between cardiovascular disorders (CVD), including hypertension, atherosclerosis (AS), and heart failure (HF), and metabolic disturbances, including insulin resistance, diabetes, and obesity, affecting 33–35% of adults and demonstrating significant associations with adverse cardiovascular events and all-cause mortality (Taha et al., 2022; Mechanick et al., 2020). According to the Global Burden of Disease (GBD) study and WHO reports, cardiometabolic diseases and related cardiovascular conditions account for more than one-third of global deaths each year, corresponding to approximately 17.9 million deaths annually, and remain the leading cause of morbidity and mortality worldwide (Dong et al., 2025; Saglietto et al., 2021). Epidemiological data indicate a continued global rise in cardiovascular disease incidence, with most patients exhibiting cardiometabolic risk factors, including obesity and dyslipidemia (Chen et al., 2025; Wang et al., 2024). China faces severe challenges, including persistently elevated cardiovascular mortality and rapidly increasing prevalence of CMD-related comorbidities (concurrent ≥2 metabolic-cardiovascular conditions), substantially worsening patient prognosis (Roth et al., 2020; In China TWCOTROCHAD and Hu, 2023). These risk factors are multifactorial, extending beyond genetic predisposition; lifestyle elements, particularly physical inactivity, smoking, and a suboptimal diet, constitute major reversible determinants (Chowdhury et al., 2023; Freisling et al., 2020). Amid accelerating population aging, the economic burden imposed by these diseases continues to escalate, presenting substantial challenges to public health systems globally (Han et al., 2021; Kivimäki et al., 2019). Despite the implementation of comprehensive prevention strategies, the global burden of CVD persists, with particularly pronounced impacts in Asia (Arena et al., 2015; Chew et al., 2023). Global life expectancy growth is projected to slow between 2016 and 2040, partially attributable to stagnating therapeutic advances in CVD management (Goh et al., 2024). Recent research has shown a strong connection between gut microbiota and the development of CMD (Yuan et al., 2024). Although most research has focused on bacteria, emerging evidence suggests that fungi play an important role. This review systematically examines current knowledge on gut mycobiome-CMD interactions, providing novel insights into future preventive and therapeutic strategies.

## 2 Methods: literature search and synthesis

This study employed a systematic literature review methodology, with comprehensive searches conducted in established databases, including PubMed, Web of Science, and the China National Knowledge Infrastructure. The core search strategy was exemplified by PubMed queries: (“gut mycobiome” OR “intestinal fungi” OR “fungal microbiome”) AND (“cardiometabolic diseases” OR “CMD” OR “atherosclerosis” OR “hypertension” OR “heart failure” OR “obesity” OR “diabetes” OR “fatty liver disease” OR “Metabolic syndrome”). During screening, priority was given to experimental or clinical studies examining gut mycobiome-CMD relationships or exploring the putative role of the mycobiome in CMD pathogenesis, which formed the basis of the inclusion criteria. Studies investigating acute infections caused by exogenous fungi leading to heart failure, as well as literature on free-living fungal organisms unrelated to the human microbiota (such as mushrooms), were excluded from this research. Subsequently, the included studies underwent rigorous evaluation to decipher how the gut mycobiome modulates CMD initiation and progression through intestinal microecological regulation.

## 3 Association between CMD and gut mycobiome

**Figure 1:**
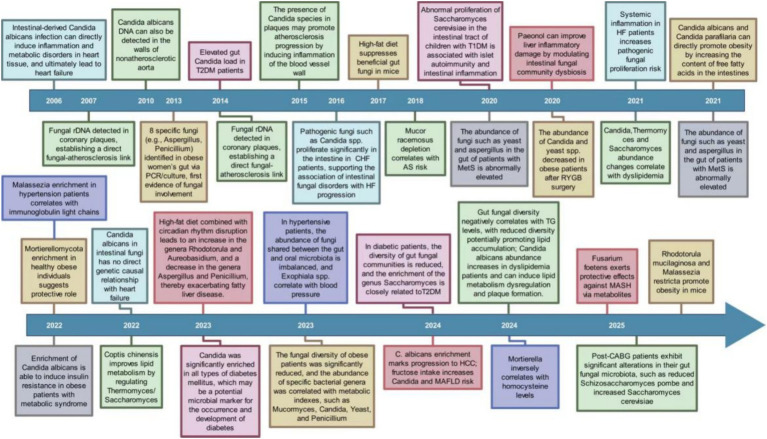
Research history of gut mycobiome and CMD (by Figdraw).

Research into how the gut mycobiome is connected to CMD was initiated in 2006. In the last twenty years, studies have progressively revealed links between the gut mycobiome and diseases such as heart failure, hypertension, atherosclerosis, diabetes, and obesity (Figure 1). These researches has evolved from detecting fungi and observing changes in their abundance to investigating their impact on disease onset and progression, while also encompassing factors like diet and intervention strategies. As the years advance, both the scope and depth of these studies continue to expand. The gut mycobiome is underscored as a crucial factor in the initiation and progression of CMD by these findings.

### 3.1 Association between obesity and gut mycobiome

Amid socioeconomic development, lifestyle transformations are driving an increased prevalence of overweight or obesity and associated health risks globally (Zhou et al., 2024). This constitutes a pressing health challenge, irrespective of the nation’s development status (Wong et al., 2020). Obesity pathogenesis involves multifaceted mechanisms, with research indicating that gut microbiota dysbiosis-induced metabolic dysregulation and functional decline may be key contributors (Geng et al., 2022). The scientific importance of the gut mycobiome (Zhan et al., 2024) is attracting increasing attention.

An early study (Gouba et al., 2013) ‌identified eight fungal genera, including *Aspergillus*, *Penicillium*, and *Malassezia*, in the gut of obese women using polymerase chain reaction amplification and fungal culture, ‌suggesting‌ a potential role for fungi in host metabolism. Subsequent studies have established the structural features of the gut mycobiome in obese individuals, revealing that fungal diversity is significantly reduced (Szóstak et al., 2023) and that alterations in specific genera are correlated with metabolic parameters.‌ Specifically, *Mucor* abundance was decreased in obese individuals (Mar Rodríguez et al., 2015), while levels of *Candida*, *Saccharomyces cerevisiae*, and *Penicillium* correlated positively with body weight, body mass index (BMI), and waist circumference (Salamati et al., 2015; Borges et al., 2018; García-Gamboa et al., 2021; Shoukat et al., 2023).‌ In an infant cohort study,‌ *Saccharomyces* ‌demonstrated potential relevance, with its abundance positively correlating with BMI and potentially predicting childhood obesity risk (Gutierrez et al., 2023). Another study found a significantly elevated phylum-level abundance of *Mortierellomycota* in metabolically healthy obese individuals, suggesting a potentially protective role (Yin et al., 2022).‌ Roux-en-Y gastric bypass, known for its effectiveness in treating obesity, can help in reestablishing mycobiome balance. Post-surgery reductions in the abundance of *Candida* and *Saccharomyces* have been observed in obese individuals (Steinert et al., 2020). *Candida species*, such as *C. albicans* and *C. parapsilosis*, contribute to obesity induced by a high-fat diet by elevating intestinal free fatty acid levels (García-Gamboa et al., 2021; Sun et al., 2021).

Animal models play a vital role in examining how the gut mycobiome is causally related to obesity. A high-fat diet impacts the gut bacterial community in mice and significantly suppresses the abundance of beneficial fungi (Heisel et al., 2017). Furthermore, antifungal treatment that disrupts the mycobiome exacerbates metabolic disorders in these models (Abdallah et al., 2020). Moreover, early colonization by specific fungi exerts a long-term impact on metabolic outcomes. Studies demonstrate that *Rhodotorula mucilaginosa* and *Malassezia restricta* promote obesity and the progression of metabolic disorders in mice (Gutierrez et al., 2025). ‌Conversely, several interventions, including a low-carbohydrate diet (Yu et al., 2022), *Litchi chinensis* seed (Xiang et al., 2022), and avenanthramide B (Liu et al., 2025), can reverse obesity progression. These interventions promote weight loss by rebalancing the ratio of beneficial to pathogenic fungi.

In summary, elevated abundance of *Candida* spp., *Saccharomyces cerevisiae*, *Penicillium* spp., *Rhodotorula mucilaginosa*, and *Malassezia restricta* may be involved in the development and onset of obesity. Conversely, *Mortierellomycota* and *Mucor* spp. may confer potential protective roles against obesity (Table 1).

**Table 1:** The role of gut mycobiome in obesity development.

Disease	Sample size	Detection method	Fungal biodiversity	Alterations of fungal composition	Reference
Obesity	27 obese patients and 12 healthy controls	Multitag Pyrosequencing	Reduce	*Mucor* ↓	Mar Rodríguez et al. (2015)
Obesity	24 eutrophic、24 overweight, 24 obese individuals	18S rDNA sequencing	Reduce	*Saccharomyces cerevisiae* ↑ *Penicillium* ↑	Borges et al. (2018)
Obesity	34 overweight, 34 obese patients and 31 healthy controls	MALDI-TOF technique	–	*C. albicans* ↑ *Rhodotorula mucilaginosa* ↑ *Saccharomyces cerevisiae* ↑	García-Gamboa et al. (2021)
Obesity	75 overweight patients and 50 healthy controls	Microbiological techniques and pathogenic profiling	Increase	*C. kefyr* ↑ *Teunomyces krusei* ↑	Shoukat et al. (2023)
Obesity	Cohort Study: 100 infants	16S rDNA and ITS2 rRNA gene sequencing	Reduce	*Saccharomyces cerevisiae* ↑ *Candida*↓ *Malassezia*↓ *Mycosphaerella*↓	Gutierrez et al. (2023)
Obesity /PCOS	PCOS patients with normal/overweight individuals	16S rRNA and ITS2 gene sequencing	Reduce	*Mortierellomycota*↓ *Candida* ↑ *Malassezia* ↑	Yin et al. (2022)
Obesity	16 morbidly obese patients/treatment of RYGB Surgical	16S rRNA gene and ITS sequencing	Reduce	*Saccharomyces cerevisiae*↓ *Candida*↓ *Pichia* ↑	Steinert et al. (2020)
Obesity	obese patients and healthy controls	qPCR	–	*C. Parapsilosiss* ↑ *C. albicans* ↑	(Sun et al. (2021))
Obesity	High-Fat Diet mice	ITS2 gene sequencing	Reduce	*Saccharomyces cerevisiae*↓	Heisel et al. (2017)
Obesity	Gnotobiotic mice fed standard and high-fat-high-sucrose diets	16S rRNA sequencing and qPCR	–	*Rhodotorula mucilaginosa* ↑ *Malassezia restricta* ↑	Gutierrez et al. (2025)

### 3.2 Association between diabetes and gut mycobiome

Diabetes mellitus is identified as a metabolic disorder with either an absolute or relative shortage of insulin and/or resistance to insulin in the organs it affects (American Diabetes Association, 2014). It can lead to various vascular and peripheral neuropathic complications, severely impairing the quality of life of affected individuals worldwide (Ahmad et al., 2022). Established risk factors, including genetics, family history, and metabolic dysregulation, have been demonstrated to play substantial roles in the development and progression of diabetes (Yu et al., 2024). ‌Recent research has elucidated a close association between gut microbiota and diabetes (Gurung et al., 2020; Zhou et al., 2022). A landmark study published in Science demonstrated that early-life exposure to *C. dubliniensis* during infancy confers protection against diabetes development (Hill et al., 2025). This study utilized human infant fecal samples to colonize murine models, revealing that mice colonized with samples from infants aged 7–12 months exhibited significantly elevated insulin expression in pancreatic islets and increased circulating serum insulin levels. Subsequent mechanistic investigations demonstrated that cell wall components derived from fungi such as *Aspergillus dublinensis* induce infiltration of islet-resident macrophages (IRMs) and promote *β*-cell regeneration in diabetic murine models, thereby attenuating diabetes incidence (Hill et al., 2025). These results further scientific insights into the involvement of the gut mycobiome in diabetes development.

Previous studies have consistently documented reduced diversity in gut fungal communities among individuals with type 2 diabetes mellitus (T2DM) (Yan et al., 2024; Gou et al., 2024; Van Syoc et al., 2024). This dysbiotic pattern is markedly exacerbated in patients with diabetic complications such as diabetic foot ulcers (Cai et al., 2024). A parallel reduction in mycobiome diversity has been observed in the cohorts of type 1 diabetes (T1DM) and gestational diabetes mellitus (GDM) (Bandala-Sanchez et al., 2022; Vavreckova et al., 2022). This evidence indicates that gut mycobiome dysbiosis, characterized by diminished diversity, is a common pathophysiological feature across subtypes of diabetes mellitus. Investigations into signature fungal taxa have revealed significant enrichment of the genus *Candida* across all major forms of diabetes mellitus. Studies have demonstrated significantly elevated intestinal *Candida* burden in patients with T2DM compared to healthy controls (Bao et al., 2023; Saleem et al., 2022; Jayasudha et al., 2020; Al Bataineh et al., 2021; Bhute et al., 2017; Rodrigues et al., 2019; Gosiewski et al., 2014). Further reinforcing this observation, research indicates that microbial translocation occurs between oral and gut microbiomes in diabetic populations (Pacheco-Yanes et al., 2023), correspondingly increasing *Candida* abundance in the oral cavity (Mussi et al., 2022). ‌Animal model studies have further demonstrated increased intestinal *Candida* colonization in diabetic dogs (Jaffey et al., 2022) and mice (Zhang et al., 2025). Moreover, *Candida* colonization rates are significantly higher in patients with T1DM (Honkanen et al., 2020; Gürsoy et al., 2018; Soyucen et al., 2014) and gestational diabetes (Vavreckova et al., 2022) than in healthy subjects. These findings suggest that aberrant *Candida* proliferation may represent a potential microbial marker for the pathogenesis of diabetes mellitus. *Yeasts* have also demonstrated significant potential in diabetic populations. The intestinal abundance of *Rhodotorula mucilaginosa* is closely associated with disease progression in T2DM studies (Cai et al., 2024), while alterations in *Saccharomycetes* class abundance correlate with the response to metformin treatment in patients with T2DM (Van Syoc et al., 2024). Cohort studies have revealed that *Saccharomyces* enrichment strengthens the association between conventional risk factors (obesity and insulin resistance) and T2DM, suggesting its potential as a predictive biomarker (Gou et al., 2024). In GDM research, changes in the abundance of taxa, such as *Hanseniaspora*, directly correlate with dysglycemia (Wu et al., 2022). Conversely, late-pregnancy intestinal enrichment of *Saccharomyces cerevisiae* in T1DM mothers frequently coincides with elevated inflammatory markers (Bandala-Sanchez et al., 2022). Similarly, aberrant *Saccharomyces* proliferation in children with T1DM is associated with pancreatic autoimmunity and intestinal inflammation. These findings further implicate *Saccharomycetes* in the pathogenesis of diabetes (Honkanen et al., 2020). ‌Furthermore, elevated intestinal levels of *Fusarium* and *Malassezia furfur* in patients with T2DM underscore the disease-specific signatures within the mycobiome. Regarding interventions, 12-week exercise training ameliorated insulin resistance by enriching beneficial fungi such as *Verticillium* (Wang et al., 2024). Additionally, Echinacoside suppresses hepatic gluconeogenesis by remodeling bacterial-fungal interaction networks (Fan et al., 2025), while exogenous H2S improves metabolism by reducing the abundance of *Candida* and *Aspergillus* (Liu et al., 2022). These approaches provide novel therapeutic avenues for precise interventions.

‌Reduced gut mycobiome diversity and increased abundance of genera, including *Candida*, *Saccharomyces*, and *Hanseniaspora*, may contribute to diabetes progression, whereas exercise-induced enrichment of *Verticillium* demonstrates potentially protective roles in diabetes amelioration (Table 2).

**Table 2:** The role of gut mycobiome in diabetes development.

Disease	Sample size	Detection method	Fungal biodiversity	Alterations of fungal composition	Reference
TD2M	Cohort Study: 12,641 individuals	ITS2 sequencing	Reduce	*Saccharomyces cerevisiae* ↑	Gou et al. (2024)
TD2M	T2DM patients/ treatment of metformin	Metagenomic sequencing	Reduce	*Saccharomycetes* ↑	Van Syoc et al. (2024)
TD2M /DF	33 T2DM patients, 32 T2DM-DF patients and 32 healthy controls	18S rDNA sequencing	Reduce	*Rhodotorula mucilaginos*↓ *Candida* ↑	Cai et al. (2024)
TD1M	45 TD1M pregnant women and 25 healthy pregnant women	ITS1 amplicon sequencing	Reduce	*Saccharomyces cerevisiae* ↑	Bandala-Sanchez et al. (2022)
GDM	29 GDM1, 31 GDM2, 22 GDM3 pregnant women and 22 healthy pregnant women	ITS1 genes sequencing	–	*Candida* ↑	Vavreckova et al. (2022)
T2DM	11 T2DM patients and 6 healthy controls	ITS2 sequencing and RT-PCR	Significant difference	*C. albicans* ↑	Bao et al. (2023)
				*Saccharomyces cerevisiae* ↑	
T2DM	21 T2DM patients and 30 healthy controls	Illumina sequencing of ITS2 region	Significant difference	*Candida* ↑	Jayasudha et al. (2020)
T2DM	25 T2DM patients and 25 healthy controls	16S rRNA and ITS2 gene sequencing	No significant difference	*Candida* ↑	Al Bataineh et al. (2021)
TD2M	14 New-DMs patients and 16 Known-DMs patients and 19 healthy controls	16S rDNA sequencing and	No significant difference	*Candida* ↑	Bhute et al. (2017)
		ITS1genes sequencing			
Diabetes	27 T1DM, 17 T2DM patients and 17 healthy controls	qPCR	–	*Candida* ↑	Gosiewski et al. (2014)
T2DM	16 T2DM patients and 13 healthy controls	Saliva and swab culture and exfoliative cytology	–	*Candida* ↑	Mussi et al. (2022)
Diabetes	14 diabetic dogs and 14 healthy control dogs	MALDI-TOF-MS	–	*Candida* ↑ *Aspergillus* ↓	Jaffey et al. (2022)
Diabetes	leptin receptor-deficient mice/ treatment of hydrogen sulfide	ITS sequencing	No significant difference	*Candida* ↓ *Aspergillus* ↓	Zhang et al. (2025)
T1DM	52 children with HLA-conferred susceptibility to T1D	ITS2 sequencing	Reduce	*Candida* ↑	Honkanen et al. (2020)
TD1M	42 TD1M patients and 42 healthy controls	Stool cultures	-	*C. albicans* ↑	Gürsoy et al. (2018)
TD1M	35 TD1M patients and 35 healthy controls	Stool cultures	Significant difference	*Bifidobacterium*↓ *C. albicans* ↑	Soyucen et al. (2014)
GDM	23 GDM pregnant women and 26 healthy pregnant women	ITS1 rDNA sequencing	Reduce	*Hanseniaspora* ↑ *Candida* ↑ *Penicillium* ↑	Wu et al. (2022)
Diabetes	39 overweight males with prediabetes/12-week exercise training	ITS2 sequencing	Increase	*Verticillium* ↑ *Iodophanus* ↑ *Monosporascus* ↑ *Bipolaris* ↑ *Conocybe* ↑	Wang et al. (2024)
T2DM	T2MD mouse model/ treatment of Echinacoside	16S rDNA and ITS sequencing	Increase	*Debarymoyces*↓ *Penicillium*↓ *Wallemia*↓	Fan et al. (2025)

### 3.3 Association between AS and gut mycobiome

‌AS is a prevalent condition that poses a substantial threat to human health, with coronary artery disease as a primary manifestation. A hallmark feature of AS lesions is the subendothelial accumulation of lipids within specific arterial regions, accompanied by smooth muscle cell proliferation and deposition of fibrous matrix components, resulting in atherosclerotic plaque formation (Shao et al., 2024). When AS affects coronary arteries, complete vascular occlusion can occur, potentially triggering myocardial infarction. Similarly, obstructions in cerebral vessels can cause cerebral infarction, commonly termed as a stroke. This pathological process constitutes the fundamental pathological basis of ischemic cardiocerebrovascular diseases (Lu and Daugherty, 2015). AS pathogenesis involves multifactorial complexity. Previous studies indicate that relevant theories primarily involve inflammation (Wolf and Ley, 2019), lipid accumulation (Bäck et al., 2019), oxidative stress (Batty et al., 2022), and endothelial injury; however, no single theory fully accounts for the entire pathogenesis of AS. ‌Beyond the established risk factors, hypertension, diabetes, hypercholesterolemia, smoking, stressors, and environmental determinants significantly accelerate lesion progression (Libby, 2021). Epidemiological data have confirmed that AS is a principal contributor to global cardiovascular mortality and morbidity. In Western populations, AS-related deaths constitute approximately 50% of all cardiovascular fatalities (Ramji and Davies, 2015). Consequently, elucidating AS pathogenesis and developing targeted therapeutic strategies have substantial clinical and scientific implications.

‌Early investigations have established a direct link between fungi and AS. In 2007, a study employing fungal rDNA detection within coronary plaques initially identified mycotic components in atherosclerotic lesions (Ott et al., 2007). Subsequent research further identified detectable *C. albicans* DNA even in non-atherosclerotic aortic walls, with a significant correlation with vascular inflammation marker expression (Jegier et al., 2010). Masoumi and Nurgeldiyeva et al. substantiated the presence of *Candida* spp. within plaques and proposed their potential role in promoting atherogenesis by inducing vascular inflammation (Masoumi et al., 2015; Nurgeldiyeva et al., 2014). These findings provide the initial evidence of fungal involvement in the pathological progression of AS.

Recent research has increasingly focused on the interplay between the gut mycobiome and host lipid metabolism dysregulation, as well as its impact on atherogenesis. Empirical evidence reveals an inverse correlation between gut fungal diversity indices and triglyceride (TG) levels, suggesting that diminished mycobiotic diversity may contribute to pathological lipid accumulation (Estrada et al., 2024). ‌Alterations in the abundance of intestinal genera, including *Candida*, *Thermomyces*, and *Saccharomyces*, correlated significantly with lipid metabolism dysregulation (Mims et al., 2021). *C. albicans* has emerged as a primary pathogenic driver, exhibiting an elevated abundance in patients with dyslipidemia. Research has demonstrated that its metabolic byproduct, formyl-methionine, provokes aberrant lipid metabolism and promotes plaque development (Wang et al., 2024). Furthermore, patients with atherosclerotic CVD exhibited significantly increased abundances of the genera *Exophiala* and *Malassezia* (Su et al., 2025). However, contradictory findings have emerged in patients with coronary heart disease, where a decreased abundance of these fungal taxa has been observed (Xu et al., 2020). This suggests that a structural imbalance in the gut mycobiome may potentiate AS progression, concurrently underscoring the importance of further clinical validation. Abundances of *Mucor racemosus* (Chacón et al., 2018) and *Fusarium* spp. (An et al., 2024) reduced significantly, demonstrating negative associations with AS risk, thereby suggesting their therapeutic potential as protective fungal taxa. ‌In clinical intervention studies, patients undergoing coronary artery bypass grafting altered significantly gut mycobiome, characterized by decreased *Schizosaccharomyces pombe* and increased *Saccharomyces cerevisiae* abundance. These shifts suggest that surgical intervention may modulate fungal communities and influence postoperative prognosis (Fu et al., 2025). Regarding pharmacological interventions, Coptis chinensis ameliorates lipid metabolism disorders by modulating *Thermomyces* spp. and *Saccharomyces* spp., revealing its dual-targeting potential toward gut bacteria and fungi (Yang et al., 2022). This provides preliminary evidence for future fungus-targeted therapeutics.

Intestinal *Candida* spp., *Thermomyces* spp., and *Saccharomyces* spp. may constitute key drivers of AS progression, whereas *Mucor racemosus* and *Fusarium* spp. may represent protective fungal taxa (Table 3).

**Table 3:** The role of gut mycobiome in ‌AS development.

Disease	Sample size	Detection method	Fungal biodiversity	Alterations of fungal composition	Reference
CAD	40 CAD patients and 20 AS patients	PCR	–	*C. albicans* ↑	Jegier et al. (2010)
Obesity	standard diet fed mice /processed diet mice	ITS2 rDNA genes sequencing	Reduce	*Candida* ↑ *Thermomyces* ↑ *Saccharomyces* ↑	Mims et al. (2021)
AS	30 dyslipidemiapatients and 30 healthy controls	ITS sequencing and qPCR	Increase	*C. albicans* ↑	Wang et al. (2024)
ACVD	214 ACVD patients and 171 healthy controls	Metagenomic sequencing	Increase	*C. albicans* ↑ *Exophiala* ↑ *Malassezia* ↑ *Penicillium Wallemia* ↑	Su et al. (2025)
CHD	24 CHD patients and 24 healthy controls	Illumina sequencing	No significant difference	*Exophiala*↓ *Malassezia*↓ *Thermoascus*↓	Xu et al. (2020)
CAD	101 CAD patients and 31 healthy controls	ITS1 rDNA genes sequencing	No significant difference	*Mucor racemosus*↓ *Fusarium*↓ *Mortierellomycota*↓	An et al. (2024)
CHD	40 CHD patients/ treatment of Coronary artery bypass grafting	Metagenomic sequencing	Reduce	*Candida* ↑ *Saccharomyces* ↑ *Schizosaccharomyces*↓	Fu et al. (2025)
Hyperlipidemia	high fat diet -induced mice/ treatment of Coptidis Rhizoma	ITS2 sequencing	Reduce	*Penicillium*↓ *Aspergillus*↓	Yang et al. (2022)

### 3.4 Association between hypertension and gut mycobiome

Hypertension is a cardiovascular syndrome characterized by sustained elevation of systemic arterial pressure. This condition poses significant risks for a spectrum of cardiocerebrovascular diseases, including stroke, coronary heart disease, and HF. According to the first Global Report on Hypertension released by the World Health Organization in 2023, data from 2019 revealed that 33% of the global population aged 30–79 years had hypertension. Approximately 10 million deaths annually are attributable to elevated systolic pressure (Kario et al., 2024). Studies have confirmed that overweight, obesity, dyslipidemia, and insulin resistance are established risk factors for hypertension (Tang et al., 2022; Guo et al., 2025), with metabolic disorders contributing to over half of hypertension cases while directly promoting vascular dysfunction and blood pressure elevation (Zhu et al., 2016).

Emerging research has revealed that the gut mycobiome mediates metabolic dysregulation and elevates blood pressure. Despite the absence of statistically significant differences in gut mycobiome diversity between hypertensive patients and healthy controls (Qiu et al., 2023; Zou et al., 2022), specific alterations in fungal abundance are significantly associated with blood pressure regulation. *Malassezia* demonstrated significant enrichment in hypertensive cohorts, and its abundance positively correlated with serum immunoglobulin light chain (*κ*/*λ*) concentrations. This implicates its pathogenic role in hypertension via immune activation (Zou et al., 2022). Conversely, *Mortierella* prevails predominantly in normotensive populations (Zou et al., 2022), presenting an inverse association with homocysteine, an established hypertension biomarker, suggesting a protective function through metabolic modulation (An et al., 2024). Several species of *Exophiala* (*E. xenobiotica* and *E. mesophila*) demonstrated direct positive correlations with systolic and diastolic blood pressure levels. Concurrently, patients with hypertension exhibit dysbiosis in shared fungal communities across gut-oral axes (Chen et al., 2023). Furthermore, the genera *Nakaseomyces* and *Saccharomyces* are specifically associated with diastolic pressure (Qiu et al., 2023).

‌The genera *Malassezia*, *Exophiala*, *Nakaseomyces*, and *Saccharomyces* demonstrate pathophysiological contributions to blood pressure elevation, whereas *Mortierella* potentially confers protective cardiometabolic benefits (Table 4).

**Table 4:** The role of gut mycobiome in hypertension development.

Disease	Sample size	Detection method	Fungal biodiversity	Alterations of fungal composition	Reference
HTN/CKD	50 HTN patients, 50 HTN + CKD patients and 50 healthy controls	ITS sequencing	Increase	*Candida*↓ *Saccharomyces* ↑ *Apiotrichum* ↑	Qiu et al. (2023)
HTN	38 pre-HTN patients, 46 HTN patients and 34 healthy controls	ITS rRNA gene sequencing	Increase	*Malassezia* ↑ *Mortierella*↓	Zou et al. (2022)
HTN	36 HTN patients and 24 healthy controls	Metagenomic sequencing	Increase	*Exophiala* ↑	Chen et al. (2023)

### 3.5 Association between HF and gut mycobiome

‌HF is a clinical syndrome primarily driven by structural and/or functional cardiac abnormalities. This pathophysiological cascade elevates the intracardiac pressures at rest or during exertion and/or compromises cardiac output, manifesting as diverse clinical symptoms (McDonagh et al., 2021). HF is an end-stage manifestation of CVD, including hypertension, and is intrinsically linked to cardiac decompensation. Despite prolonged investigations into the association between HF progression and gut microbiota alterations (Sandek et al., 2007), a definitive causal link remains unestablished (Tang et al., 2019).

A prior Mendelian randomization analysis revealed no genetic causal relationship between *C. albicans* gut colonization and HF pathogenesis (Luo et al., 2022), whereas discordant conclusions have emerged from independent cohort studies. Animal studies have demonstrated that in C5-deficient mice, gut-derived *C. albicans* infection directly triggers cardiac inflammatory damage and metabolic dysregulation, ultimately culminating in heart failure (Mullick et al., 2006). Clinical investigations have revealed a significant proliferation of pathogenic fungi, including *Candida* spp., in the gut mycobiome of patients with chronic heart failure (CHF), concomitant with elevated intestinal permeability and systemic inflammation. These alterations are positively correlated with disease severity, further supporting the association between gut mycobiome dysbiosis and HF progression (Pasini et al., 2016). Moreover, heightened inflammatory cytokine levels in CHF may compromise immune function and increase susceptibility to pathogenic fungal overgrowth (García-Torre et al., 2021). This provides evidence of a complex, mutually reinforcing relationship between HF and gut mycobiome perturbations involving immune competence, medication exposure, and inflammatory status. Therapeutically, targeted probiotic supplementation (*Saccharomyces boulardii*) failed to improve cardiac function or microbial diversity in the GutHeart trial (Awoyemi et al., 2021), indicating complexity in microbiota modulation strategies.

Gut mycobiota, particularly *Candida* spp., could be crucial in the beginning and advancement of HF (Table 5). However, current evidence remains limited, warranting further in-depth investigations.

**Table 5:** The role of gut mycobiome in HF development.

Disease	Sample size	Detection method	Fungal biodiversity	Alterations of fungal composition	Reference
CHF	60 CHF patients and 20 healthy controls	Stool cultures	–	*Candida* ↓	Pasini et al. (2016)
Saccharomyces boulardii in HF	Multicenter randomized clinical trial:151 patients	18S rDNA sequencing and PCR	No significant difference	*No significant change*	Awoyemi et al. (2021)

### 3.6 Association between metabolic syndrome (MetS) and gut mycobiome

‌MetS is a pathological state characterized by dysregulated metabolism of carbohydrates, lipids, and proteins. Its four core features are obesity, dyslipidemia, hepatic steatosis, and insulin resistance (Ndumele et al., 2023). Globally, MetS affects approximately 25% of the population and contributes to two-thirds of noncommunicable disease-related mortality, and its prevalence continues to rise worldwide (Saklayen, 2018). Evidence suggests an association between MetS and gut mycobiome dysbiosis.

Previous clinical investigations have demonstrated that the enrichment of *C. albicans* in the gut induces insulin resistance in obese patients with MetS, potentially serving as a key mediator linking metabolic dysregulation and immune imbalance (Nikolic et al., 2022). Further studies revealed that vitamin D3 deficiency may drive opportunistic fungal overgrowth, including *Candida* spp., thereby increasing the susceptibility of patients with psoriasis to MetS (Marchlewicz et al., 2024). This association is more pronounced in pregnant women, whose MetS correlates with gut microbial dysbiosis characterized by overgrowth of pathogenic fungi (*C. albicans*) and reduced abundance of *Bifidobacterium* and *Lactobacillus* (Pavlovska et al., 2020). Additionally, elevated gut fungal burdens of *Saccharomyces* and *Aspergillus* spp. have been documented in patients with MetS (Gradisteanu Pircalabioru et al., 2022). In parallel, dysregulation of adipokines constitutes another central feature of MetS. Pro-inflammatory adipokines such as TNF-*α*, IL-6, leptin, and resistin are upregulated, whereas anti-inflammatory adipokines including adiponectin and omentin are downregulated. This imbalance drives chronic low-grade inflammation, insulin resistance, and vascular dysfunction. Emerging evidence suggests that gut fungal dysbiosis, particularly Candida overgrowth, may exacerbate this adipokine imbalance by promoting systemic inflammation and thereby worsening metabolic and cardiovascular complications (Datta et al., 2025). ‌Regarding probiotic fungal interventions, the administration of *Saccharomyces boulardii* improves insulin sensitivity and ameliorates lipid metabolic disorders in patients with MetS by suppressing pathogenic fungal overgrowth (Egea et al., 2023).

The evidence suggests that *C. albicans* might play a vital role in the pathogenesis of MetS; however, the implications of *Saccharomyces* and *Aspergillus* spp. require further mechanistic substantiation (Table 6).

**Table 6:** The role of gut mycobiome in MetS development.

Disease	Sample size	Detection method	Fungal biodiversity	Alterations of fungal composition	Reference
MetS	38 MS patients	Stool cultures	–	*C. albicans* ↑	Nikolic et al. (2022)
MetS	52 MS pregnant women and 25 healthy pregnant women	Stool cultures	Reduce	*C. albicans* ↑ *Bifidobacterium* ↓ *Lactobacillus*↓	Pavlovska et al. (2020)
MetS	30 MS patients and 30 healthy controls	18S rDNA sequencing and qRT-PCR	–	*Candida* ↑ *Aspergillus* ↑	Gradisteanu Pircalabioru et al. (2022)

### 3.7 Association between metabolic dysfunction-associated fatty liver disease (MAFLD) and gut mycobiome

MAFLD is a chronic metabolic stress-related liver injury in genetically predisposed individuals, primarily driven by overnutrition and insulin resistance (Targher et al., 2024). Currently, the most prevalent chronic liver disorder has historically been termed non-alcoholic fatty liver disease (NAFLD) (Eslam et al., 2020). It has been redefined to emphasize its pathogenic links to T2DM dysregulation, involving multifaceted factors such as obesity and T2DM (Jurek et al., 2025). The growing exploration of gut microbial ecosystems further implicates mycobiome dysbiosis in MAFLD pathogenesis.

‌Accumulating evidence suggests that intestinal mycobiome dysbiosis occurs in patients with MAFLD. Fungal compositional analyses demonstrated an elevated abundance of multiple fungal taxa in NAFLD-affected guts, alterations that may drive hepatic injury, dysregulated lipid metabolism, and disease progression (You et al., 2021). Dysbiosis within the genus *Candida* is particularly prominent, with *C. albicans* and *C. krusei* significantly increased in MAFLD cohorts (Demir et al., 2022; Zhang et al., 2024; Hrncir et al., 2024). The increased abundance of *C. albicans* further serves as a biomarker for progression from fatty liver or cirrhosis to early-stage hepatocellular carcinoma (Jiang et al., 2024). Intestinal *Pichia kudriavzevii* and *Candida* spp. have been demonstrated to endogenously generate ethanol and TG via fructose-dependent pathways, directly contributing to non-alcoholic steatohepatitis pathogenesis (Mbaye et al., 2022). Concurrently, the abundance of genus *Mucor* is correlated with hepatic inflammation and fibrosis, with the subspecies *Mucor ambiguus* demonstrating significant associations with obesity, dyslipidemia, and liver injury biomarkers (Demir et al., 2022; Viebahn et al., 2024; Niu et al., 2023). Environmental and dietary risk factors further exacerbate mycobiome dysbiosis. For instance, a high-fat diet combined with chronodisruption (chronic jet lag) disrupts microbial circadian oscillations, elevates *Rhodotorula* and *Cyphellophora* spp., and reduces *Aspergillus* and *Penicillium* spp., thereby aggravating fatty liver pathology (Zheng et al., 2023). ‌Moreover, high-fructose intake amplifies the abundance of *Candida* spp. by remodeling bacterial-fungal cross-kingdom networks, mediating host metabolic dysfunction, and elevating the MAFLD risk (Hrncir et al., 2024). Therapeutically, the intestinal fungus *Fusarium foetens* exerts hepatoprotective effects against metabolic dysfunction-associated steatohepatitis by secreting bioactive secondary metabolites (Zhou et al., 2025). Concurrently, the phytochemical paeonol demonstrates therapeutic potential by ameliorating histopathological injury by remolding mycobiome dysbiosis (Wu et al., 2020; Ge et al., 2021).

‌In summary, *Candida* spp., *Mucor* spp., *Pichia kudriavzevii*, and other *Candida yeasts* correlated positively with MAFLD progression, whereas *Fusarium foetens* exhibited protective potential (Table 7).

**Table 7:** The role of gut mycobiome in MAFLD development.

Disease	Sample size	Detection method	fungal biodiversity	Alterations of fungal composition	Reference
NAFLD	79 NAFLD patients and 34 healthy controls	ITS2 sequencing	Increase	*Talaromyces* ↑ *Paraphaeosphaeria* ↑ *Lycoperdon* ↑ *Curvularia* ↑ *Leptosphaeria*↓ *Pseudopithomyces*↓ *Fusicolla*↓	You et al. (2021)
NAFLD	78 NAFLD patients, 16 controls and 73 patients with alcohol use disorder	Songbird	No significant difference	*C. albicans* ↑ *C. krusei* ↑	Demir et al. (2022)
ALD/ MAFLD	48 ALD patients, 55 MAFLD patients and 64 healthy controls	ITS2 sequencing	No significant difference	*C. albicans* ↑ *Malassezia restricta* ↑	Zhang et al. (2024)
MASLD	A human microbiota-associated mouse model /treatment of fructose with or without preservatives	ITS sequencing	Reduce	*Candida* ↑ *Penicillium* ↑	Hrncir et al. (2024)
Primary liver disease	39 patients: 6 HCC, 14 LC, 8 CH, 11 AH	ITS sequencing	Reduce	*C. albicans* ↑	Jiang et al. (2024)
NASH	10 NASH patients and 10 healthy controls	Stool cultures	–	*Candida* ↑	Mbaye et al. (2022)
ALD/ NAFLD	48 ALD patients and 78 NAFLD patients	ITS2 sequencing	Increase	*Mucor racemosus* ↑	Viebahn et al. (2024)
MAFLD	21 MAFLD patients and 20 healthy controls	Metagenomic sequencing	Reduce	*Mucor ambiguus* ↑ *Saccharomyces cerevisiae*↓	(Niu et al. (2023))
MAFLD	High fat and high fructose diet fed mice/ treatment of chronic jet lag	ITS sequencing	Reduce	*Rhodotorula* ↑ *Cyphellophora* ↑ *Aspergillus*↓ *Penicillium*↓	Zheng et al. (2023)

## 4 Gut mycobiome-mediated mechanisms in CMD progression

**Figure 2:**
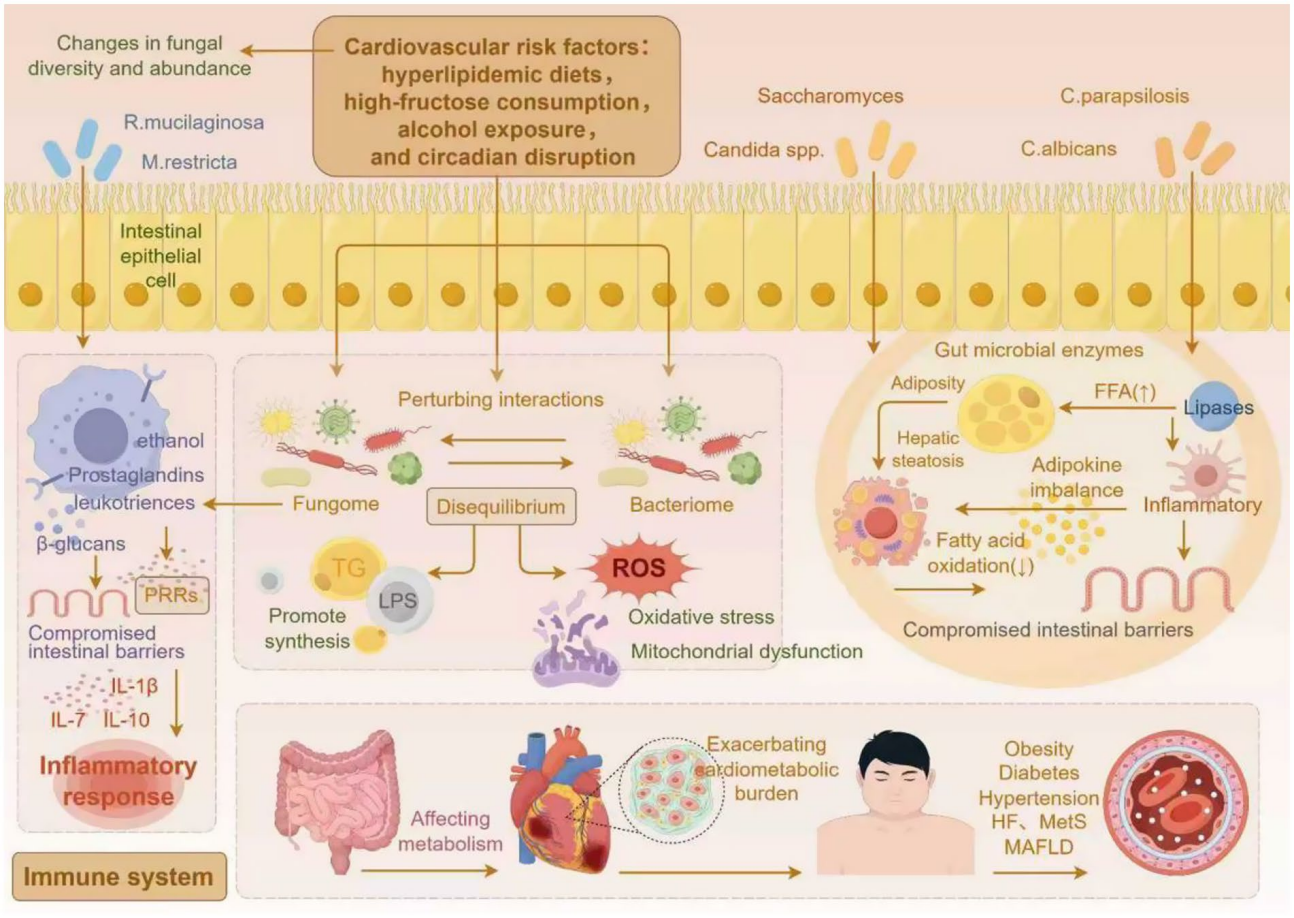
The gut mycobiome modulates cardiometabolic disease progression via associated mechanisms (by Figdraw).

The gut mycobiome participates in the onset and progression of cardiometabolic diseases through multiple mechanisms. Existing evidence suggests that it may contribute to disease progression via mechanisms such as the release of metabolites, cross-kingdom interactions, and the immune-metabolic axis (Figure 2).

### 4.1 Gut mycobiome-derived metabolites promote lipid absorption and accumulation

The gut mycobiome directly participates in host metabolism and modulates physiological processes through its derived metabolites. Gut microbial enzymes represent an emerging research focus, as they catalyze metabolic reactions that enhance the breakdown and absorption of nutrients (Jiang et al., 2024). Recent evidence has indicated that the gut microbiota and their enzymatic derivatives significantly drive the progression of T2DM (Wang et al., 2023) and MAFLD (Luo et al., 2025). *Candida parapsilosis* secretes lipases that hydrolyze dietary fats, thereby elevating circulating FFA concentrations (Sun et al., 2021). FFA accumulation promotes obesity through multiple pathways, primarily by inducing inflammation that disrupts intestinal barrier integrity, secondarily by enhancing energy absorption and surplus in the gut, and ultimately by activating fat-storage pathways while inhibiting fatty acid oxidation, thereby promoting adiposity and hepatic steatosis, collectively exacerbating cardiometabolic burden (Sun et al., 2021). Furthermore, excessive fructose intake has been definitively linked to an elevated risk of CMD (Zhang et al., 2020; Soleimani et al., 2023; Drożdż et al., 2021). High-fructose diets alter bacterial-fungal cross-kingdom interactions, significantly enriching *Candida* spp. and *Saccharomyces* (Hrncir et al., 2024). These proliferating gut fungi metabolize fructose to ethanol while driving *de novo* TG synthesis. Both endogenously generated ethanol and TG accelerate MASLD development (Mbaye et al., 2022).

### 4.2 Gut mycobiome-bacterial community cross-kingdom interaction dysbiosis and its mechanistic implications

Research has established that the intestinal fungome and bacteriome maintain homeodynamic equilibrium through reciprocal modulation, collectively preserving gut ecosystem stability (Underhill and Iliev, 2014; Elnagar, 2024). This interaction mainly manifests as competition for nutrients, co-metabolism of dietary components, or modulation of the host immune system (Santus et al., 2021). Moreover, gut fungi can inhibit bacterial colonization. For example, the presence of *Candida albicans* reduces the efficacy of fecal microbiota transplantation (FMT) in treating Clostridioides difficile infection, and further studies have shown that *Candida albicans* suppresses bacterial engraftment (Zuo et al., 2018). Conversely, gut bacteria can also inhibit fungal proliferation; for instance, colonization by Lactobacillus has been shown to suppress the expansion of Candida (Strus et al., 2005). Moreover, gut fungi and bacteria not only antagonize each other, but also cooperate to promote pathogenesis. For example, *Escherichia coli* enhances the invasion of *Candida albicans* into intestinal epithelium in an *in vitro* model (Yang et al., 2016); another study showed that monocolonization with gut fungi alone does not induce physiological changes in germ-free mice, whereas co-colonization with fungi and bacteria significantly exacerbates colonic inflammation. These findings further highlight that the interaction between fungi and bacteria is fundamental to the onset and progression of disease (Zhang et al., 2022; Krüger et al., 2019). It is well known that diet and lifestyle, as major modifiable factors of CMD, are among the most effective regulators of microbial community interactions (Guiducci et al., 2023). Studies show that a high-fat diet is associated with increased abundance of *Candida* and decreased abundance of *Prevotella* (Heisel et al., 2017; Peroumal et al., 2022). High-carbohydrate diets show a positive correlation with elevated abundances of *Candida* and *Methanobrevibacter* (Hoffmann et al., 2013). Furthermore, high-fat, high-fructose diets (HFFD) increase the abundance of pathogenic fungi (*Aspergillus*, *Penicillium*, and *Candida*), while reducing the abundance of bacterial taxa including *Lachnospiraceae_NK4A136_group* and *Harryflintia* (Zhao et al., 2024; Zhao et al., 2025). Moreover, many of these bacteria with reduced abundance are probiotics capable of producing short-chain fatty acids (SCFAs), and the association between decreased SCFA levels and CMD has been extensively studied and well established (Neves et al., 2015; Wang et al., 2025). These findings confirm that diet-induced alterations in fungal and bacterial communities are closely associated with host metabolic dysregulation (glucose and lipid metabolism) and vascular inflammatory phenotypes (Zhao et al., 2024; Zhao et al., 2025). Furthermore, circadian disruption, an emerging cardiometabolic risk factor, worsens hepatic inflammation and lipid metabolism dysregulation by perturbing gut mycobiota-bacteriome interactions during chronic jetlag (Zheng et al., 2023). These findings indicate that dysregulation of the fungal-bacterial interaction network impairs microbial regulation of host energy harvest and immune homeostasis, thereby triggering inflammation due to intestinal barrier defects, as well as abnormalities in short-chain fatty acid and bile acid metabolism (Wang et al., 2020), ultimately driving the pathological progression of CMD. Further fungal transplantation experiments have reinforced this understanding. For instance, single gavage administration of *Candida albicans* increased the relative abundance of *Staphylococcus* and *Mucispirillum*, while decreasing the abundance of *Akkermansia*. *Staphylococcus* may produce LPS, which can enter the bloodstream through a compromised intestinal mucosal barrier, thereby triggering chronic low-grade inflammation—a key mediator in the development of insulin resistance, diabetes, obesity, and atherosclerosis (Bao et al., 2023). Furthermore, multiple studies have confirmed that supplementation with *Akkermansia* can ameliorate cardiometabolic diseases (Gofron et al., 2024; Niu et al., 2024), while a reduction in *Akkermansia* abundance further exacerbates disease pathogenesis. Although existing evidence supports the synergistic role of opportunistic fungal proliferation alongside harmful bacterial overgrowth and beneficial bacterial suppression in the pathogenesis of cardiometabolic diseases, it remains unclear whether gut fungi and bacteria also modulate CMD progression through antagonistic interactions—a question that represents a promising avenue for future research.

### 4.3 Gut mycobiome-immune-metabolic axis crosstalk mechanisms

Gut mycobiome drives metabolic dysregulation by modulating the immune system. First, gut fungal-derived metabolites (including candidalysin, prostaglandins, formyl-methionine [Wang et al., 2024), leukotrienes, and ethanol (Santelmann and Howard, 2005; Noverr et al., 2002; Tan et al., 2019)] can induce inflammatory responses. For example, candidalysin is a fungal peptide toxin secreted by *Candida albicans* that directly induces IL-1α, IL-1*β*, IL-8, IL-36, and the NOD-, LRR-, and pyrin domain-containing protein 3 (NLRP3) inflammasome (Zhou et al., 2024). These inflammatory mediators are known to play critical roles in the development of insulin resistance, T2DM, obesity, vascular endothelial injury, atherosclerosis, and NAFLD (Li et al., 2025). Prostaglandins (PGs) are a class of eicosanoids, comprising homologs from series 1, 2, and 3. Studies indicate that gut fungi such as *Cryptococcus neoformans* and *Candida albicans* can secrete prostaglandins via *de novo* synthesis or through the conversion of exogenous arachidonic acid (Noverr et al., 2001). Among these, PGE₂ has been most extensively studied and participates in regulating diverse physiological and pathological processes, associated with diseases such as diabetes, hypertension, obesity, non-alcoholic fatty liver disease, and cardiovascular diseases (Wang et al., 2021). On one hand, PGE₂ binds to the EP3 receptor to activate extracellular signal-regulated kinase 1/2 (ERK1/2), thereby inducing serine phosphorylation of insulin receptor substrate 1 (IRS1) and ultimately exacerbating insulin resistance (Henkel et al., 2009). On the other hand, PGE₂ induces β-cell dysfunction via the IL-1β-mediated NF-κB activation pathway and promotes hepatic lipid accumulation through NF-κB signaling (Chung et al., 2015). In summary, PGE₂ effectively mediates NF-κB pathway activation and dysregulation of glucose and lipid metabolism, triggering a cascade of pathological alterations in vascular endothelium (Xu et al., 2020), liver, and adipose tissue, thereby driving the progression of cardiometabolic diseases. Formyl-methionine is a metabolite that can be produced by *Candida albicans* (Wang et al., 2024). Studies show that the concentration of formyl-methionine in circulation is closely associated with the risk of various cardiovascular diseases (Cai et al., 2021). This substance can activate the HIF-2α signaling pathway in the gut, thereby promoting ceramide synthesis and ultimately accelerating the progression of atherosclerosis (Wang et al., 2024). Previous studies have demonstrated that ceramide, an emerging compound, is closely associated with CMD (Wilkerson et al., 2024). This bioactive lipid can induce insulin resistance, oxidative stress, and atherosclerotic plaque progression (Spaggiari et al., 2024). Secondly, gut fungi can also induce inflammatory responses through cell wall components. Upon recognition by host pattern recognition receptors (PRRs), their cell wall components (chitin, mannans, and *β*-glucans) trigger inflammatory cascades that exacerbate metabolic disorders (Cheng et al., 2011; Gringhuis et al., 2012). For instance, *Rhodotorula mucilaginosa* and *Malassezia restricta*, when recognized by host PRRs, drive pathological lipid accumulation by inducing intestinal low-grade inflammation and enhancing nutrient uptake, thereby promoting high-fat diet-induced obesity (Gutierrez et al., 2025). Fungal β-glucans translocate across damaged intestinal barriers, bind to the CLEC7A receptor on Kupffer cells, and trigger IL-1β-dependent hepatic inflammation and fibrosis (Yang et al., 2017), subsequently accelerating the progression of steatohepatitis. Similarly, in mouse models, *Candida albicans*-derived β-glucans released via the dectin-1 receptor activate inflammatory cascades to induce insulin resistance; conversely, eliminating *C. albicans* or blocking this pathway significantly alleviates insulin resistance and halts the progression of metabolic disorders (Bao et al., 2023). The aforementioned mechanistic studies reinforce the causal link between gut fungi and CMD. Gut fungal derivatives promote the pathogenesis and progression of metabolic cardiovascular disorders via immunoinflammatory mechanisms, as well as through pathways inducing insulin resistance and lipid metabolic disorders. Furthermore, the roles of gut bacterial derivatives—such as bile acids, LPS, and trimethylamine N-oxide (TMAO)—in CMD have been well-established (Kondapalli et al., 2025; Du et al., 2025). However, whether metabolites derived from bacteria and fungi exert synergistic or antagonistic effects in CMD remains unclear, and their interactive mechanisms warrant further elucidation in future studies.

## 5 Therapeutic strategies targeting the gut mycobiome

**Figure 3:**
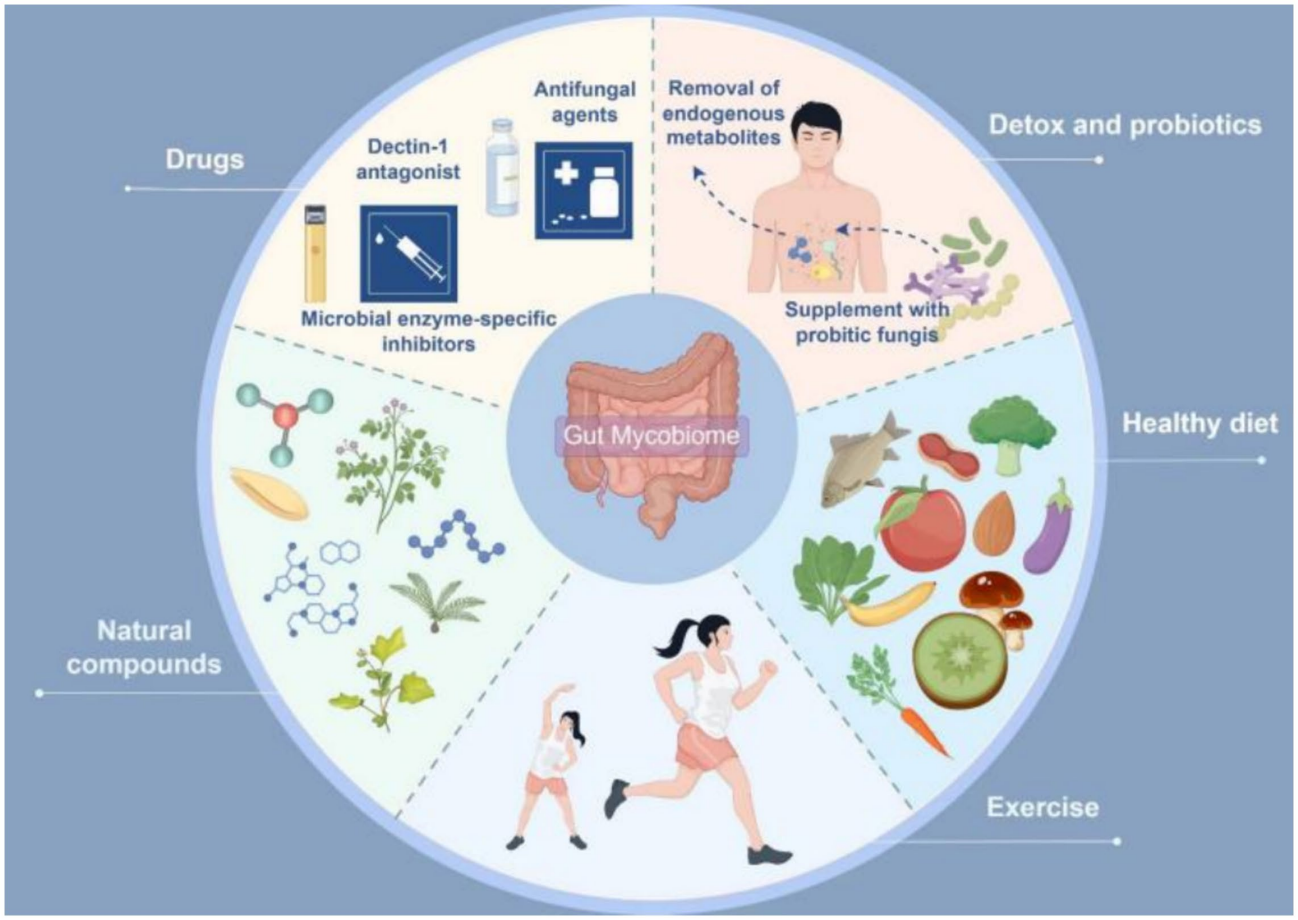
Interventions targeting the gut mycobiome in cardiometabolic diseases (by Figdraw).

The gut mycobiome is closely associated with the onset and progression of cardiometabolic diseases. Recent evidence suggests that multiple potential drugs or therapies can modulate the progression of CMD by regulating the gut mycobiota (Table 8). Therefore, exploring intervention strategies based on the gut mycobiome may offer novel approaches for treating cardiometabolic disorders. Current methods for modulating the gut mycobiome can be broadly categorized into two main types: targeted therapies against gut fungi and their derivatives, and comprehensive modulation of the gut mycobiome-immune-metabolism axis. Specific interventions include: antifungal agents, microbial enzyme-specific inhibitors, clearance of endogenous metabolites, probiotic fungal supplementation, Dectin-1 antagonists, dietary interventions (Mediterranean diet, low-carbohydrate diet, high-fiber diet), natural compounds, and physical exercise (Figure 3).

**Table 8:** Potential drugs or therapies for treating CMD by modulating the gut mycobiota.

Intervention method	Study type	Efficacy	Safety	Mechanism	Disease	Fungi	Ref
Low-Carb Diet	Clinical Study	Significant weight loss; marked improvement in blood lipid and glucose levels	No serious adverse events reported	Extensive covariation between gut fungi and bacteria may indirectly affect host metabolism	Obesity	Significant increase in fungal diversity	Yu et al. (2022)
*Litchi chinensis* seed	Preclinical Study	Reduced body weight in obese zebrafish; improved fat accumulation and lipid metabolism	No serious adverse events reported in the study	Modulation of gut microbiota and mycobiota composition improves obesity	Obesity	Regulated proportions of fungi with specific metabolic capabilities, including *Penicillium* and *Candida*	Xiang et al. (2022)
12-Week High-Intensity Interval Training	Clinical Study	Improved insulin sensitivity in the exercise group; reduced fasting insulin and blood lipid levels	No serious adverse events reported	Exercise-induced dynamic changes in gut fungi are closely associated with metabolic improvements	Diabetes	Significant increase in fungal diversity; effectively reshaped fungal community composition	Mussi et al. (2022)
Echinacoside (ECH)	Preclinical Study	Reduced fasting blood glucose, improved insulin resistance, alleviated hyperlipidemia	No adverse reactions explicitly reported	Suppressed expression of key gluconeogenic enzymes (FBP1, PCK1, G6PC) in the liver by regulating bacterial-fungal transkingdom network	T2DM	Decreased: *Debaryomyces* Increased: Wallemia, Penicillium, Aspergillus, Cladosporium, Alternaria	Jaffey et al. (2022)
NaHS	Preclinical Study	Reduced serum triglycerides, blood glucose, and serum insulin levels; improved obesity	No adverse reactions explicitly reported	Improved metabolic disorders by modulating gut bacterial and fungal community structure; serum H₂S levels correlated with abundance of specific genera	T2DM	Decreased: *Candida, Aspergillus*	Zhang et al. (2025)
Coptidis Rhizoma extract	Preclinical Study	Reduced body weight, body fat, and blood lipids; alleviated hepatic lipid accumulation;	No adverse reactions explicitly reported	Improved lipid metabolism by modulating gut bacterial and fungal composition and their interactions	Hyperlipidemia	Increased: *Aspergillus ruber, Mortierella kuhlmanii, Tilletta bromi* Decreased: *Aspergillus chevalieri, Mortierella alpina*	Yang et al. (2022)
Rifaximin or Saccharomyces boulardii	Clinical Study	No significant efficacy observed	Well-tolerated	—	HFrEF	Without significant effects	Awoyemi et al. (2021)
Fusarium foetens	Preclinical Study	Improved hepatic steatosis, inflammation, and fibrosis; reduced liver weight and blood lipid levels	Good safety profile	Altered ceramide metabolism by inhibiting CerS6, a key enzyme in the ceramide biosynthesis pathway, thereby ameliorating MASH progression	MASH	Increased: *Fusarium foetens*	Demir et al. (2022)
Paeonol	Preclinical Study	Reduced blood lipid levels, improved liver function, alleviated hepatic steatosis	No adverse reactions explicitly reported	Reduced translocation of 1,3-β-D-glucan (produced by intestinal fungi, especially *Candida albicans*) to the liver	Alcoholic Liver Disease	Reduced abundance of pathogenic fungi such as *Candida albicans*; improved fungal dysbiosis	Zhang et al. (2024)

### 5.1 Therapeutic approaches targeting gut fungi and their metabolites

Targeted modulation of gut fungi and their derivatives holds therapeutic promise for the integrative management of cardio-metabolic diseases. First, previous studies have demonstrated that pharmacological ablation of *C. albicans* using conventional antifungals (amphotericin B or fluconazole) significantly ameliorates insulin resistance and metabolic derangements (Wang et al., 2024). Fluconazole monotherapy also exhibits promising efficacy in attenuating AS progression by suppressing *C. albicans* proliferation, which concurrently curbs systemic inflammation and ceramide accumulation, thereby reducing cardiometabolic risk (Bao et al., 2023). Given that amphotericin B and fluconazole are broad-spectrum antifungal agents whose long-term use may incur significant side effects, whether antifungal therapy should be implemented as a potential clinical intervention for cardiometabolic diseases thus requires further clinical investigation and evidence-based validation. Second, targeted inhibition of microbial enzymatic activity is a promising therapeutic strategy. The groundbreaking discovery of microbial DPP4 isozymes has unveiled a novel mechanism (Wu et al., 2023). Consequently, developing specific inhibitors against fungal enzymes, such as *C. parapsilosis* lipase, can attenuate excessive FFA production, thereby ameliorating oxidative lipotoxicity and hepatic steatosis (Sun et al., 2021); this has significant therapeutic implications for CMD amelioration. ‌Furthermore, the depletion of fungal-derived metabolites (endogenous ethanol or ceramides) confers distinct physiological benefits. Notably, ceramides, designated as the ‘putative second cholesterol’ (Zhang et al., 2025), render pharmacological modulation of their biosynthetic pathways, such as serine palmitoyltransferase (SPT), a novel therapeutic paradigm for CMD management. Consequently, Myriocin (thermozymocidin), a potent SPT inhibitor, demonstrates substantial translational promise for mitigating CMDs (Gengatharan et al., 2025).

### 5.2 Integrated modulation of gut mycobiome-immune-metabolic axis

‌As previously indicated, fungal β-glucan, a prototypical pathogen-associated molecular pattern, serves as a master regulator of host immunity. Its binding to Dectin-1 receptors on immune cells primarily instigates inflammatory cascades (Liu et al., 2025). Pioneering studies have suggested β-glucan-specific nanobodies hold therapeutic promise owing to their target-blocking capacity (Liu et al., 2023). Developing Dectin-1 antagonists is a viable therapeutic alternative (Li et al., 2024) to mitigate inflammation-driven metabolic derangements. Moreover, dysbiosis within the gut mycobiome-bacteriome interactome fuels cardiometabolic pathogenesis via hub-mediated immuno-metabolic axes. ‌Accumulating clinical evidence supports the therapeutic efficacy of fungal-targeted transplantation or supplementation with probiotic fungi. Nevertheless, cutting-edge findings suggest that dietary modulation is the linchpin for reconfiguring gut microbiota, surpassing conventional microbiota transfer (Kennedy et al., 2025). Established paradigms, including Mediterranean, low-carbohydrate, and high-fiber diets, demonstrate a documented potential to correct metabolic derangements (Gao et al., 2024). Additionally, natural compounds constitute an indispensable therapeutic arsenal against CMD. Previous studies have demonstrated that *Litchi chinensis* seed (Xiang et al., 2022), echinacoside (Fan et al., 2025), Coptidis Rhizoma (Yang et al., 2022), and paeonol (Wu et al., 2020) exhibit dual lipid-glycemic modulatory efficacy, recalibrating fungal-bacterial interactomes to remodel gut ecological architectures. Furthermore, the indisputably beneficial role of exercise serves as a vital adjunct strategy for weight management (Jung et al., 2015), mitigation of metabolic dysregulation (Wewege et al., 2018), and cardiovascular health enhancement (Yue et al., 2022; Ramos et al., 2015). Contemporary research reveals that compared to sedentary counterparts, a 12-week regimen of high-intensity interval training significantly augmented the abundance of gut mycobiota including Verticillium, Ceratocystis, and Sarocladium. This elevation in fungal populations demonstrated significant correlation with exercise-induced improvements in key metabolic parameters—namely blood glucose levels, blood pressure, and lipid profiles (Wang et al., 2024).

## 6 Limitations

Despite the growing body of evidence linking the gut mycobiome to CMD, several important limitations must be acknowledged. First, the majority of existing studies are cross-sectional or conducted with small cohorts, which limits the generalizability and reproducibility of the findings. Large-scale, longitudinal, and multi-center studies are still lacking, making it difficult to draw firm conclusions about temporal dynamics and causal relationships. Second, most current evidence remains correlative. Although associations between specific fungal taxa (e.g., *Candida* and *Saccharomyces*) and CMD have been observed, direct mechanistic and causal links are insufficiently established. Experimental validation, particularly *in vivo* models and interventional trials, is urgently needed to confirm the pathogenic or protective roles of fungi in metabolic and cardiovascular pathways. Third, methodological and technical constraints remain a significant barrier. Current high-throughput sequencing techniques often lack sufficient depth and taxonomic resolution for fungi compared to bacteria. For example, 18S rRNA gene sequencing is widely employed for eukaryotic classification, yet it demonstrates limited resolution at the fungal species level, particularly in samples with highly diverse microbial communities (e.g., gut microbiota) (Hibbett et al., 2007; Jansen et al., 2024). In contrast, Internal Transcribed Spacer (ITS) sequencing—especially targeting the ITS1 and ITS2 regions—has become the predominant approach for fungal identification and diversity analysis in the gut (Diaz and Dongari-Bagtzoglou, 2021). However, the ITS region exhibits substantial length variation, which can introduce PCR amplification bias. Furthermore, its analytical outcomes are heavily dependent on reference sequence databases and cannot distinguish between viable and non-viable organisms. These factors may compromise identification accuracy; therefore, primer selection requires particular caution (Schoch et al., 2012; Nilsson et al., 2009). Secondly, in fine-scale species-level classification, shotgun metagenomic sequencing theoretically offers superior accuracy compared to ITS sequencing, as it provides access to more comprehensive genomic information, including functional genes. Despite its potential in mycobiome research, shotgun metagenomics faces significant challenges in gut fungal analysis: fungi typically constitute a minor fraction of the gut microbiota, with their extremely low DNA abundance often overshadowed by overwhelming bacterial DNA (Huseyin et al., 2017; Thomas et al., 2012; Xie et al., 2023). Furthermore, while emerging technologies such as MALDI-TOF and DART mass spectrometry have been explored for fungal strain classification, their utility and robustness in holistically profiling complex gut mycobiota—particularly regarding comprehensive taxonomic resolution and functional characterization—require rigorous validation and optimization (Neurauter et al., 2025). Furthermore, fungal cultivation is challenging due to slow growth rates and complex nutritional requirements, which hinders species-level characterization and functional analyses. Improvements in sequencing coverage, primer design, and fungal-specific bioinformatics pipelines are required to advance the field. Finally, translation into clinical practice remains limited. While preliminary evidence suggests that antifungal therapies, probiotic fungal supplementation, and dietary interventions may hold therapeutic potential, clinical studies validating these approaches are scarce. The safety, efficacy, and long-term effects of gut mycobiome-targeted therapies require rigorous evaluation before clinical application can be realized. Taken together, these limitations underscore that our current understanding of gut mycobiome–CMD interactions remains in its infancy. Addressing these challenges will be crucial for transforming correlative insights into mechanistic understanding and developing clinically applicable diagnostic and therapeutic strategies.

## 7 Conclusion and perspectives

This is the first systematic review to explore the link between the gut mycobiome and CMD. By incorporating mycobiota evidence into CMD pathogenesis models, we provide a comprehensive overview of the latest progress in this field. In contrast to previous reviews that primarily focused on bacterial microbiota-CMD correlations, this systematic demonstration reveals the impact of intestinal fungi on disease progression via multiple pathways, including the secretion of metabolites and the regulation of the immune-metabolic axis. This study formulates a new research framework by integrating advanced findings from metagenomic and clinical cohort studies, moving beyond traditional bacteria-centric strategies. This holistic perspective highlights previously undervalued therapeutic targets within the fungal microbiota and creates opportunities for novel CMD intervention strategies. Therefore, dietary modulation, supplementation with probiotic fungi, natural compounds, and targeted therapies against the immune-metabolic axis, when synergistically combined with conventional pharmacotherapies, may represent key strategies for the effective management of cardiometabolic diseases in the future.

In future studies, clinical validation of fungal biomarkers in CMD remains pivotal for translational applications. Accumulating evidence suggests elevated abundances of the genera Candida and Saccharomyces in CMD, encompassing diabetes, obesity, and atherosclerosis (AS). However, large-scale cohort validation is urgently required. Future research should deploy multicenter, prospective cohorts to authenticate fungal signatures and integrate mycobiome biomarkers with key clinical indices (BMI, glycemia, lipidemia, and hypertension) to establish precision diagnostic matrices for CMD. These efforts will catalyze the development of mechanism-based antifungal therapeutic pipelines. Moreover, cardiometabolic-protective fungal consortia, exemplified by Mortierella spp. and Mucor racemosus, which demonstrate significant inverse associations with CMD pathogenesis, warrant mechanistic dissection. Emerging evidence posits that therapeutic modulation of the gut mycobiota equilibrium may constitute the definitive frontier for next-generation metabolic therapeutics.

To address this gap, advancing from association to causality requires deepening mechanistic understanding of gut mycobiome-CMD crosstalk. This research nexus remains nascent, yet existing studies delineate Candida -dominated fungal consortia that orchestrate metabolic reprogramming through effector metabolites and cell wall components. The precise pathological cascades by which fungal-derived molecules perturb distal organ homeostasis via spatiotemporal circulatory trafficking demand systematic deconvolution. Integrated metabolomic, proteomic, and single-cell topological mapping will be essential to unravel these mechanisms, bridging the gap between observational data and causal inference. Multi-omics technologies, as a recently emerged integrative biomedical tool, provide a novel framework for elucidating the complexity of the gut microbiome (Chetty and Blekhman, 2024). Metagenomics enables precise classification of gut fungal strains and assessment of the potential functions of the fungal community through DNA profiling; metaproteomics identifies key proteins and their contributions to host biochemical pathways via protein expression profiling of gut fungi and the host; metatranscriptomics reveals which genes are actively expressed under specific conditions by RNA expression profiling, thereby elucidating the actual functional state of gut fungi; and metabolomics detects small-molecule metabolites generated from the interaction between gut fungi and the host (Duan et al., 2025). When analyzing multi-omics characteristics of the gut mycobiome, host multi-omics data and phenotypic data (e.g., diet, lifestyle habits, clinical indicators) must be concurrently collected. For instance, the FoCus cohort study integrated multi-omics data across phenomics, microbiome, metabolome, genome, metagenome, and nutritional profiling levels to systematically investigate the stepwise progression of CMD from healthy status to overt metabolic dysregulation and ultimately to cardiovascular disease (Geisler et al., 2022). The multi-omics testing generates vast amounts of data, and effectively analyzing and interpreting these complex biological insights has become the central challenge in current research (Rozera et al., 2025). Although a consensus on optimal integration strategies has not yet been reached, existing approaches—such as dimensionality reduction and clustering analysis, correlation analysis, Bayesian networks, machine learning, and AI model development—can be leveraged for application (Chetty and Blekhman, 2024; Wu et al., 2024). For example, a multi-omics study investigating the mechanism by which exercise ameliorates type 2 diabetes effectively integrated gut mycobiome, metabolome, and proteome data through correlation analysis, revealing gut mycobiome-mediated metabolic changes (Wang et al., 2024). Another study performed integrated analysis of multi-omics data for NAFLD using Bayesian networks (Chella Krishnan et al., 2018). Additionally, researchers can leverage newly developed software packages to integrate multi-omics data; for instance, in the cardiometabolic disease cohort (MetaCardis), the tool successfully revealed associations between the microbiome and metabolome (Muller et al., 2024). Furthermore, by integrating Mendelian randomization (MR) analysis, multi-omics data—including the gut mycobiome—can be systematically assessed for potential causal relationships with CMD (He et al., 2024). Comprehensive application of these strategies enables effective integration of multi-omics data, thereby facilitating a deeper understanding of the complex role of the gut mycobiome in CMD.

Moreover, disease prevention during critical early-life windows presents a promising avenue for long-term CMD risk reduction and should be a focus of future research and public health strategies. During the birthing process, infants acquire maternal fungal communities through contact with the vaginal canal and maternal skin (Willis et al., 2019), as well as through postnatal skin-to-skin contact (Heisel et al., 2019; Schei et al., 2017) or breastfeeding (Schei et al., 2017). Research indicates vertical transmission of these fungal communities from mother to infant: when specific fungal DNA is present in the mother’s gut, the probability of detecting corresponding fungal DNA in the infant’s gut significantly increases (Boutin et al., 2021; Fujimura et al., 2016). The diversity of the infant gastrointestinal fungal community markedly expands during the first year of life, with dominant genera undergoing stage-specific shifts (Boutin et al., 2021; Fujimura et al., 2016). Specifically, Candida predominates at birth; by 6 months of age, Malassezia and Cystofilobasidium gradually become dominant; and by 18 months, Trichosporon emerges as the primary genus (Zeng et al., 2025). The early establishment of the gut mycobiota is influenced by multiple factors. Maternal factors—including geography, environment, diet, mode of delivery, and antibiotic use—affect the composition of breast milk fungal communities. Infant factors—such as feeding practices, antibiotic exposure, and age—also shape the gut fungal composition (Rodriguez et al., 2024). Among these, mode of delivery (cesarean section) is the most extensively studied. For instance, infants or mice born via cesarean section exhibit higher risks of developing obesity and diabetes in adulthood compared to those delivered vaginally (Cox et al., 2014; Jensen et al., 2022; Keag et al., 2018). This underscores the critical importance of microbial community establishment during early-life critical windows, where the presence of specific microbial taxa contributes to optimal immune development and healthy metabolic homeostasis (Zeng et al., 2025). Landmark research identifies gut-adapted Candida dubliniensis colonization during the neonatal window as a master regulator of pancreatic *β*-cell ontogeny, establishing a groundbreaking conceptual framework for understanding diabetes pathogenesis. This discovery necessitates expanding investigations into the establishment trajectories of early-life gut mycobiota and their causal entanglement with CMD. Here, gut-fungal reprogramming emerges as a viable substrate for primordial interceptive therapeutics, offering a novel paradigm for disease prevention.

In addition to mechanistic and clinical priorities, overcoming persistent technical challenges is critical to accelerating progress in the field. Overcoming persistent technical challenges is critical to accelerating progress in the field. Notwithstanding significant advancements in gut mycobiome research, technical limitations such as inadequate sequencing coverage, limited taxonomic discrimination, challenging cultivation requirements, and low strain isolation efficiency remain unresolved. Future efforts must prioritize the development of fungal-specific primers and advanced metagenomic assembly algorithms to achieve species-level classification accuracy. Such technological evolution will expedite the translation of fundamental mycobiome discoveries into precision diagnostic and therapeutic strategies for CMD, ultimately realizing the bench-to-bedside paradigm.
